# Refractory Digital Ulcers in Systemic Sclerosis Sine Scleroderma Associated With Antiphospholipid Syndrome: A Case-Based Review

**DOI:** 10.7759/cureus.92198

**Published:** 2025-09-13

**Authors:** Inês Almeida, Liliana Saraiva, Vera Romão, João Tavares, Inês Fróis Cunha, Maura Couto

**Affiliations:** 1 Rheumatology, Unidade Local de Saúde Viseu Dão-Lafões, Viseu, PRT; 2 Internal Medicine, Unidade Local de Saúde Viseu Dão-Lafões, Viseu, PRT

**Keywords:** antiphospholipid syndrome, digital ulcers, pulmonary embolism, systemic sclerosis sine scleroderma, vasculopathy

## Abstract

Digital ulcers (DUs) are a severe manifestation of the vasculopathy underlying systemic sclerosis (SSc). In cases refractory to treatment, the presence of other modifiable causes of vasculopathy, such as antiphospholipid syndrome (APS), should be considered, although its diagnosis in patients with systemic sclerosis sine scleroderma (ssSSc) is exceedingly rare. We report the case of a 20-year-old Caucasian female with ssSSc, who presented with Raynaud’s phenomenon (RP), recurrent DU, telangiectasias, inflammatory arthralgias, dyspepsia, and epigastric pain, in the absence of skin thickening. Laboratory investigation revealed a high-titre antinuclear antibody (ANA; 1:2560) with an anti-centromere pattern, and positivity for both anti-centromere antibodies (ACA) and anti-Ro-52 antibodies. Nailfold capillaroscopy showed an early scleroderma pattern. Despite optimized vasodilatory therapy, including calcium channel blockers, phosphodiesterase inhibitors, endothelin receptor antagonists, and regular iloprost infusions, the patient continued to experience recurrent DU. Following a pulmonary embolism with lingula infarction, anticoagulation was initiated, resulting in complete healing of the DU within two months and no subsequent thrombotic events. Repeat testing confirmed persistent positivity for lupus anticoagulant (LA) and anti-β2-glycoprotein I (anti-β2GPI) IgG more than 12 weeks apart, establishing the diagnosis of secondary APS. To our knowledge, this is the fourth reported case of ssSSc with secondary APS, and the first presenting with recurrent DU and pulmonary embolism, highlighting the rarity of this association and the importance of considering APS in SSc patients with refractory DU. The association between SSc and APS remains poorly understood, and further research is needed to clarify the underlying mechanisms and guide optimal management.

## Introduction

Systemic sclerosis (SSc) is a rare connective tissue disease characterized by the association of autoimmune features, vasculopathy, and fibrosis of the skin and/or internal organs [1]. Vasculopathy is central to the pathogenesis of SSc, involving complex interactions among endothelial cells, vascular smooth muscle cells, extracellular matrix, and circulating mediators, ultimately leading to vascular remodelling, vasospasm, and vessel occlusion [2]. Raynaud’s phenomenon (RP) and digital ulcers (DUs) are manifestations of this underlying vasculopathy, with critical digital ischemia being the most feared complication [3].

Systemic sclerosis sine scleroderma (ssSSc), first described by Rodnan and Fennell [4], is characterized by the presence of SSc-associated visceral manifestations and serologic abnormalities, in the absence of skin fibrosis [5]. This subset accounts for 8.8% of all patients registered in the international EUSTAR database, where a large cohort of 350 ssSSc patients reported current or previous DU in 4.7% and 19.0% of cases, respectively, and demonstrated a predominance of anti-centromere antibodies (ACA; 61%) over antitopoisomerase antibodies (15.1%) [6]. According to the database, ssSSc patients with DU tended to be younger, had more frequent oesophageal involvement, a higher prevalence of anti-U1RNP antibodies, and elevated creatine kinase levels compared to those without DU [6]. In the presence of DU, it is important to consider the potential coexistence of other causes of vasculopathy, such as large vessel disease, embolism, inflammation, infection, and paraneoplastic syndromes, particularly in cases refractory to standard treatment [7].

Antiphospholipid syndrome (APS) is a systemic autoimmune disease characterized by arterial, venous, or microvascular thrombosis, pregnancy morbidity, or other non-thrombotic manifestations in patients with persistent antiphospholipid antibodies (aPL) [8]. APS can be classified as primary, occurring in isolation, or secondary, when associated with an underlying condition such as a connective tissue disease, malignancy, or medication exposure [9].

According to the Euro-phospholipid Project, SSc accounts for only 0.7% of APS cases [10]. The prevalence of APS among SSc patients is estimated to be approximately 4%, based on a French multicentre prospective study [11]. In patients with ssSSc, secondary APS has been rarely reported [12-14]. To the best of our knowledge, this is the first reported case of ssSSc presenting with recurrent DU and pulmonary embolism attributed to secondary APS.

## Case presentation

A 20-year-old Caucasian female was diagnosed with ssSSc at the age of 18, presenting at diagnosis with RP, multiple DU, telangiectasias, inflammatory arthralgias, dyspepsia, and epigastric pain. She denied alcohol, tobacco, or illicit drug use. On physical examination, the patient had no puffy fingers, skin thickening, or calcinosis. Laboratory findings are summarized in Table 1.

**Table 1:** Initial laboratory investigations ACA: anti-centromere antibody; aCL: anticardiolipin antibody; ANA: antinuclear antibody; anti-β2GPI: anti-β2-glycoprotein I antibody; Ig: immunoglobulin; LA: lupus anticoagulant; mL: millilitre; U: units

Investigation	Result	Reference range
ANA	1:2560, centromere pattern	<1:160
ACA	Positive	Negative
Anti-Ro-52	Positive	Negative
LA	Positive	Negative
Anti-β2GPI IgG	9.0 U/mL	0.0-10.0 U/mL
Anti-β2GPI IgM	4.0 U/mL	0.0-10.0 U/mL
aCL IgG	22.0 U/mL	0.0-40.0 U/mL
aCL IgM	5.3 U/mL	0.0-40.0 U/mL

Nailfold capillaroscopy showed an early scleroderma pattern, and upper gastrointestinal endoscopy identified mild chronic gastritis. There was no evidence of cardiac or pulmonary involvement.

Initial management included nifedipine 30 mg/day, pentoxifylline 800 mg/day, and intravenous iloprost via an elastomeric pump, yielding partial symptom control. Due to persistent DU, the nifedipine dose was increased to 60 mg/day, and sildenafil (up to 75 mg/day), bosentan (up to 250 mg/day), and acetylsalicylic acid (100 mg/day) were added. Despite optimized therapy, DU continued to recur, requiring ongoing iloprost infusions.

**Figure 1:**
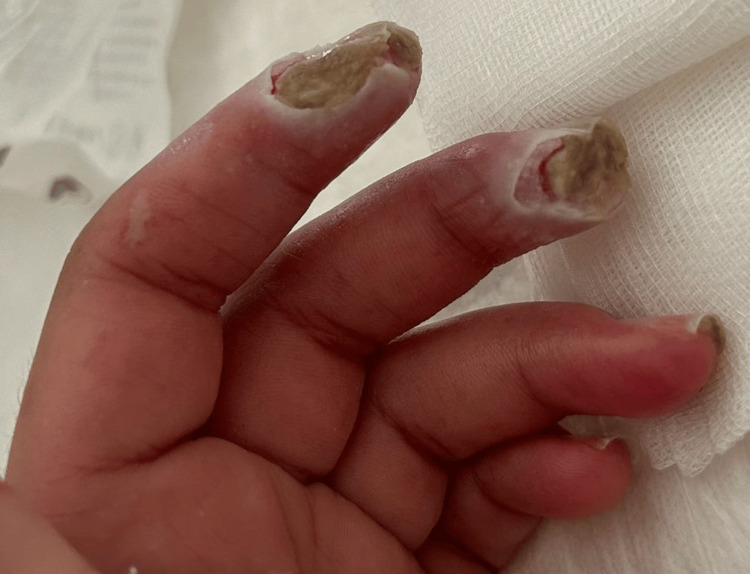
Digital ulcers on the second and third fingers of the left hand The image shows two digital ulcers in a patient with systemic sclerosis sine scleroderma, which were refractory to optimized vasodilator therapy, including calcium channel blockers, phosphodiesterase inhibitors, and regular iloprost infusions, as well as additional strategies such as endothelin receptor antagonists and acetylsalicylic acid.

The patient later presented with deep, painful DU on the pulp of the second and third fingers of the left hand, with signs of infection but with preserved distal pulses (Figure 1).

She was admitted for intravenous iloprost, antibiotics (initially trimethoprim/sulfamethoxazole, later switched to ciprofloxacin), wound care, and pain management, which required opioid analgesia. Further laboratory investigations were conducted, and the results are presented in Table 2.

**Table 2:** Laboratory investigations during the first hospitalization μL: microlitre; aCL: anticardiolipin antibody; ANCA: anti-neutrophil cytoplasmic antibodies; anti-β2GPI: anti-β2-glycoprotein I antibody; anti-MPO: anti-myeloperoxidase antibody; anti-PR3: anti-proteinase 3 antibody; aPTT: activated partial thromboplastin time; C3: complement component C3; C4: complement component C4; CRP: C-reactive protein; dL: decilitre; dsDNA: anti-double-stranded deoxyribonucleic acid antibodies; ESR: erythrocyte sedimentation rate; g: grams; h: hour; Ig: immunoglobulin; INR: international normalised ratio; LA: lupus anticoagulant; L: litre; mg: milligrams; mL: millilitre; mm: millimetres; PT: prothrombin time; U: units

Investigation	Result	Reference range
Haemoglobin	14.4 g/dL	12.0-15.0 g/dL
White blood cells	6.13 × 10^9^/L	4.50-11.50 × 10^9^/L
Neutrophils	3.3 × 10^9^/L	2.0-7.5 × 10^9^/L
Platelet count	177 × 10^9^/L	150.0-450.0 × 10^9^/L
ESR	24.0 mm/h	0.0-20.0 mm/h
CRP	0.71 mg/dL	<0.50 mg/dL
dsDNA	10.0 U/mL	0.0-15.0 U/mL
Anti-MPO	0.30 U/mL	0.0-3.0 U/mL
Anti-PR3	0.30 U/mL	0.0-3.0 U/mL
Cryoglobulins	Negative	Negative
C3	130.0 mg/dL	83.0-177.0 mg/dL
C4	17.3 mg/dL	12.0-36.0 mg/dL
IgA	195.0 mg/dL	40.0-350.0 mg/dL
IgG	1075.0 mg/dL	650.0-1600.0 mg/dL
IgM	130.0 mg/dL	50.0-300.0 mg/dL
Serum protein electrophoresis		
Albumin	4.05 g/dL	4.02-4.76 g/dL
Alpha-1 globulin	0.44 g/dL	0.21-0.35 g/dL
Alpha-2 globulin	0.96 g/dL	0.51-0.85 g/dL
Beta globulin	0.81 g/dL	0.57-0.99 g/dL
Gamma globulin	1.00 g/dL	0.80-1.35 g/dL
Total protein	7.20 g/dL	6.60-8.70 g/dL
Urinalysis		
pH	5.5	4.5-8.0
Protein	Negative	Negative
Glucose	Negative	Negative
Ketones	Negative	Negative
Bilirubin	Negative	Negative
Nitrite	Negative	Negative
White blood cells	Not detected	≤11.8/µL
Erythrocytes	Not detected	≤10.1/µL
Hyaline casts	Not detected	Usually none
Crystals	Not detected	Usually none
Protein/creatinine	0.066 mg/mg	<0.15 mg/mg
Coagulation studies		
PT	13.8 seconds	11.7-15.3 seconds
INR	1.06	0.8-1.2
aPTT	31.0 seconds	25.0-34.0 seconds
Fibrinogen	3.1 g/L	2.0-4.0 g/L
Factor V Leiden gene mutation	Not detected	Negative
Prothrombin G20210A gene mutation	Not detected	Negative
MTHFR gene mutation	Not detected	Negative
Protein C activity	99%	70.0-140.0%
Protein S activity	56%	54.7-123.7%
Antithrombin activity	111%	83.0-128.0%
LA	Positive	Negative
Anti-β2GPI IgG	74.0 U/mL	0.0-10.0 U/mL
Anti-β2GPI IgM	8.2 U/mL	0.0-10.0 U/mL
aCL IgG	25.0 U/mL	0.0-40.0 U/mL
aCL IgM	6.5 U/mL	0.0-40.0 U/mL

Colour Doppler ultrasonography showed inflammatory parietal thickening and stenosis of the left radial artery. Mycophenolate mofetil was introduced and titrated to 2 grams/day, based on the suspicion of an underlying inflammatory vasculopathy contributing to ischemia.

Approximately one month later, the patient developed pleuritic chest pain, dyspnoea, and fever, testing positive for severe acute respiratory syndrome coronavirus 2 (SARS-CoV-2). Computed tomography pulmonary angiogram revealed pulmonary thromboembolism, complicated by lingula infarction, and left-sided pleural effusion, necessitating a second hospital admission. Anticoagulation was initiated with low molecular weight heparin (1 mg/kg twice daily), later transitioned to warfarin (target international normalised ratio (INR) of 2-3), and acetylsalicylic acid (100 mg/day) was maintained. Mycophenolate was discontinued, and nirmatrelvir/ritonavir, along with ceftriaxone, were started for presumed bacterial co-infection.

Thoracocentesis of the left-sided pleural effusion yielded exudative fluid; infectious serologies and cultures (blood, urine, pleural fluid) were negative. Despite treatment, the patient developed febrile neutropenia, requiring sequential antibiotic escalation (piperacillin/tazobactam, meropenem, and vancomycin). Bone marrow aspiration confirmed a reactive process, and recovery was achieved with filgrastim. Subsequently, she developed a right pleural effusion and a new pulmonary infarction. A second thoracocentesis revealed sterile exudate. Laboratory test results from this second admission are summarised in Table 3.

**Table 3:** Laboratory investigations during the second hospitalization μL: microlitre; Ab: antibody; ADA: adenosine deaminase; Ag: antigen; ALP: alkaline phosphatase; ALT: alanine transaminase; AST: aspartate aminotransferase; CMV: cytomegalovirus; CRP: C-reactive protein; dL: decilitre; EBV: Epstein-Barr virus; ESR: erythrocyte sedimentation rate; g: grams; GGT: gamma-glutamyl transferase; h: hour; HIV: human immunodeficiency virus; Ig: immunoglobulin; IU: international units; L: litre; LDH: lactate dehydrogenase; mg: milligrams; mL: millilitre; mm: millimetres; PCR: polymerase chain reaction; SARS-CoV-2: severe acute respiratory syndrome coronavirus 2; U: units; ULN: upper limit of normal; VCA: viral capsid antigen

Investigation	Result		Reference range/interpretation
	Pre-treatment	Post-treatment	
Haemoglobin	9.4 g/dL	12.9 g/dL	12.0-15.0 g/dL
White blood cells	1.31 × 10^9^/L	4.84 × 10^9^/L	4.50-11.50 × 10^9^/L
Neutrophils	0.1 × 10^9^/L	2.3 × 10^9^/L	2.0-7.5 × 10^9^/L
Platelet count	197.0 × 10^9^/L	154.0 × 10^9^/L	150.0-450.0 × 10^9^/L
ESR	140.0 mm/h	28.0 mm/h	0.0-20.0 mm/h
CRP	8.59 mg/dL	0.74 mg/dL	< 0.50 mg/dL
Procalcitonin	0.10 ng/mL	0.04 ng/mL	0.0-0.50 ng/mL
Serum protein	5.6 g/dL	6.7 g/dL	6.6-8.7 g/dL
Serum LDH	176.9 IU/L	243.0 IU/L	120.0-246.0 IU/L
Serum albumin	3.8 g/dL	4.2 g/dL	3.5-5.0 g/dL
Glucose	82 mg/dL	88 mg/dL	74.0-106.0 mg/dL
GGT	10.5 IU/L	15.8 IU/L	0.0-38.0 IU/L
ALP	59.0 IU/L	62.0 IU/L	25.0-100.0 IU/L
AST	16.0 IU/L	18.0 IU/L	3.0-31.0 IU/L
ALT	10.0 IU/L	13.0 IU/L	3.0-31.0 IU/L
Total bilirubin	0.7 mg/dL	0.7 mg/dL	0.3-1.2 mg/dL
Urea	15.0 mg/dL	24.0 mg/dL	19.0-49.0 mg/dL
Creatinine	0.5 mg/dL	0.6 mg/dL	0.5 - 1.2 mg/dL
SARS-CoV-2 PCR	Positive		Negative
CMV IgG	137.00 U/mL		≤12.00 U/mL
CMV IgM	10.90 U/mL		≤18.00 U/mL
Parvovirus B19 IgG	<0.1 Index		<0.9 Index
Parvovirus B19 IgM	<0.1 Index		<0.9 Index
EBV VCA IgG	9.0 U/mL		<18.0 U/mL
EBV VCA IgM	5.0 U/mL		<36.0 U/mL
HIV 1/2 Ag/Ab combo	Non-reactive		Non-reactive
Aspergillus galactomannan Ag	<0.2 Index		<0.5 Index
Blood culture	Negative		No growth
Urine culture	Negative		No growth
Thoracocentesis (first procedure)			
Fluid appearance	Yellow-orange		Clear or pale yellow
pH	7.43		7.35-7.45
Protein	4.1 g/dL		Exudate (pleural fluid protein > 0.5 × serum protein)
LDH	361.0 IU/L		Exudate (pleural LDH > 0.6 × serum LDH and > 2/3 ULN)
Albumin	2.5 g/dL		Serum-effusion albumin gradient = 1.3 g/dL
ADA	32.5 U/L		0.0-40.0 U/L
Glucose	89.0 mg/dL		60-100 mg/dL
Nucleated cells	776.0/µL		<1000/µL
Differential (neutrophils/lymphocytes)	71.2/28.8		Suggests acute inflammation
Pleural fluid culture	Negative		No growth
Thoracocentesis (second procedure)			
Fluid appearance	Yellow-orange		Clear or pale yellow
pH	7.43		7.35-7.45
Protein	4.5 g/dL		Exudate (pleural fluid protein > 0.5 × serum protein)
LDH	332.0 IU/L		Exudate (pleural LDH > 0.6 × serum LDH and > 2/3 ULN)
Albumin	2.9 g/dL		Serum-effusion albumin gradient = 0.9 g/dL
ADA	38.4 U/L		0.0-40.0 U/L
Glucose	68.0 mg/dL		60-100 mg/dL
Nucleated cells	1934.0/µL		<1000/µL
Differential (neutrophils/lymphocytes)	22.1/77.9		Suggests a chronic or subacute process
Pleural fluid culture	Negative		No growth
Bone marrow aspiration			
Cellularity	Normocellular aspirate with abundant fragments		Marrow cellularity appropriate for age
Neutrophils	24% of total cells decreased to terminally mature forms		40-60% Mild maturation abnormality with arrest at the myelocyte stage
Monocytes	7% of total cells are in normal maturation		2-10% Normal
Erythroid cells	58% of total cells, hyperplasia with occasional nuclear bridges and basophilic stippling		20-30% Erythroid hyperplasia; reactive stress/accelerated erythropoiesis
Megakaryocytes	Increased cellularity, multiple maturation stages, some giant forms, no dysplastic features		Abnormal reactive megakaryopoiesis, no evidence of dysplasia or myeloproliferative disorder
Lymphocytes	8.4% of total cells: T, B, NK cells with normal phenotype		10-20% normal
Plasma cells	0.02% of total cells Normal phenotype		0-5% normal
Blasts	3.7% of total cells, 9% B-lymphoid, 15% immature, 48% granulocytic/neutrophilic, 20% erythroid, 13% monocytic/dendritic lymphoplasmacytoid		≤5% Heterogeneous blast population, below threshold for acute leukemia
Fibrosis	None		None
Iron stores	Adequate		Normal
Overall impression	Normocellular marrow with discrete maturational abnormalities across hematopoietic lineages		Findings consistent with a reactive process; no evidence of dysplasia, clonal proliferation, or hematological malignancy

**Figure 2:**
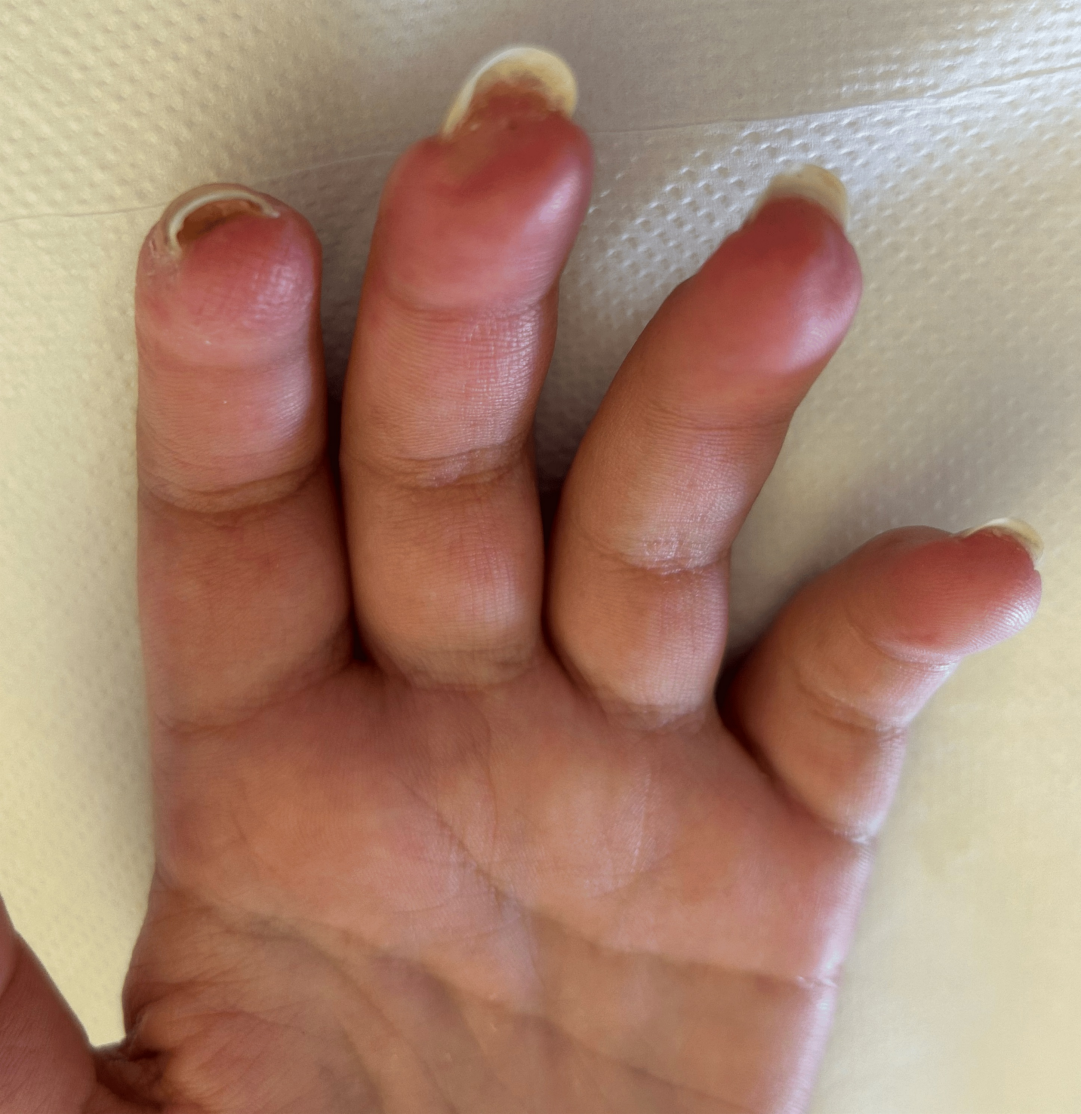
Complete healing of the digital ulcers following two months of anticoagulation therapy Following the diagnosis of secondary antiphospholipid syndrome, anticoagulation was initiated, first with low molecular weight heparin and later transitioned to warfarin. The image shows complete healing of the digital ulcers on the second and third fingers of the left hand two months after the initiation of this therapy.

After 23 days of broad-spectrum antibiotics, persistent fever, negative cultures, and unremarkable imaging (including positron emission tomography/computed tomography), a non-infectious inflammatory process was assumed. Prednisolone at a dose of 0.5 mg/kg/day was initiated and gradually tapered, leading to sustained clinical and laboratory improvement. Following two months of anticoagulation, the DU healed completely (Figure 2).

Over 24 months of follow-up, the patient experienced four minor, isolated, and uncomplicated DU episodes, likely related to difficulty in maintaining the target INR; these resolved quickly with local wound care. No additional thrombotic events, bleeding events, or new organ involvement were observed. Repeat testing confirmed persistent positivity for lupus anticoagulant (LA) and anti-β2-glycoprotein I (anti-β2GPI) IgG antibodies (Table 4), measured more than 12 weeks apart, establishing the diagnosis of secondary APS.

**Table 4:** Reassessment of antiphospholipid antibodies These results were obtained more than 12 weeks apart from the measurements presented in Table 2. aCL: anticardiolipin antibody; anti-β2GPI: anti-β2-glycoprotein I antibody; LA: lupus anticoagulant; mL: millilitre; U: units

Investigation	Result	Reference range
LA	Positive	Negative
Anti-β2GPI IgG	59.0 U/mL	0.0-10.0 U/mL
Anti-β2GPI IgM	9.0 U/mL	0.0-10.0 U/mL
aCL IgG	19.0 U/mL	0.0-40.0 U/mL
aCL IgM	7.4 U/mL	0.0-40.0 U/mL

**Figure 3:**
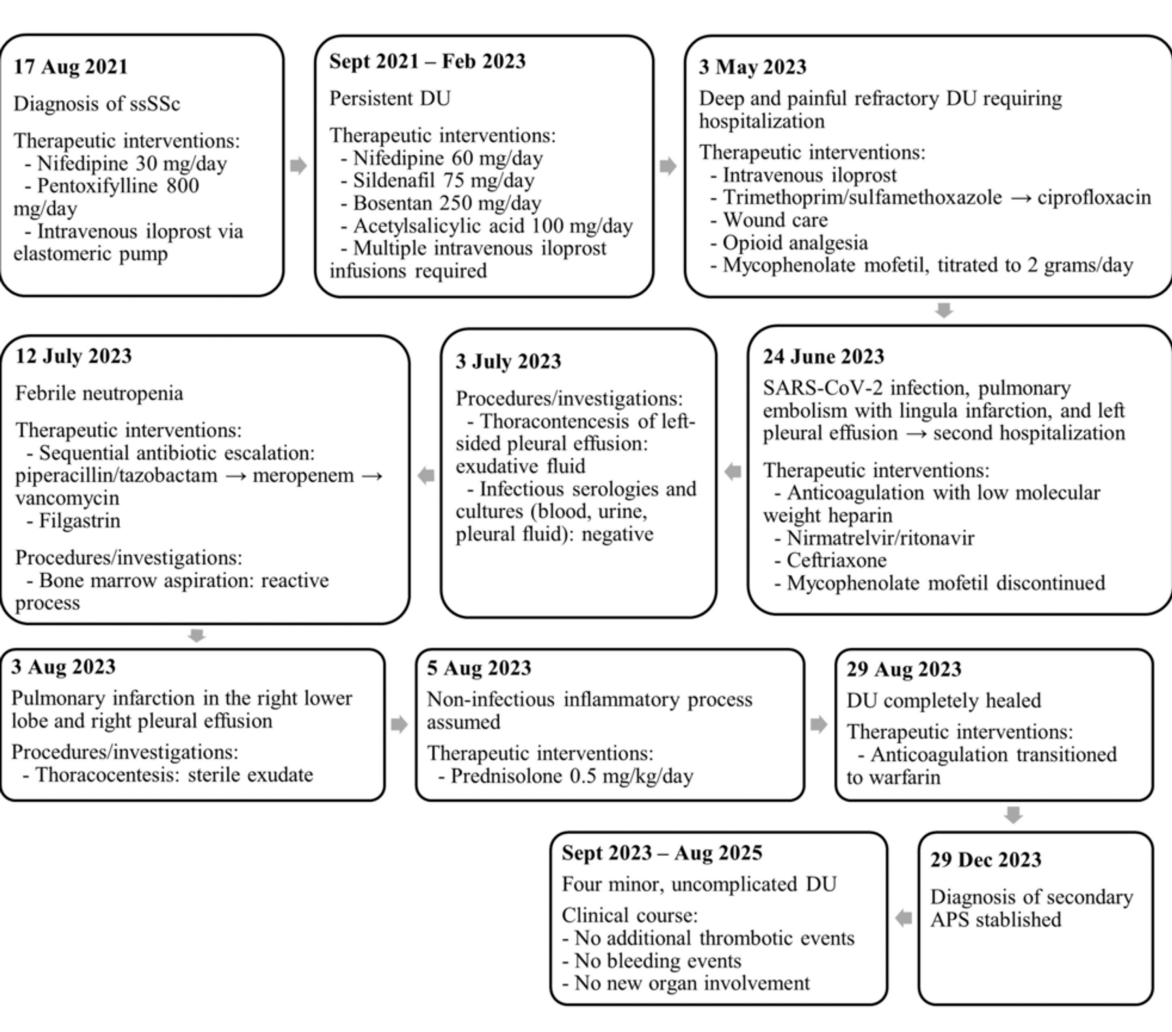
Timeline of major clinical events The figure summarizes the patient’s clinical course from diagnosis in August 2021 through August 2025, highlighting the timing of diagnoses, hospitalizations, thrombotic events, therapeutic interventions, and outcomes, including complete digital ulcer healing and long-term follow-up without new organ involvement. APS: antiphospholipid syndrome; DU: digital ulcers; kg: kilogram; mg: milligrams; ssSSc: systemic sclerosis sine scleroderma

A timeline summarizing the patient’s key clinical events is shown in Figure 3.

## Discussion

We describe a case of ssSSc associated with secondary APS, which, to our knowledge, is the fourth reported case of this clinical association, highlighting its rarity and clinical relevance. Notably, this is the first case to manifest both severe digital ulceration and pulmonary embolism.

The previously reported cases are summarized below. Key clinical characteristics of all cases, including ours, are presented in Table 5.

**Table 5:** Clinical characteristics of patients with systemic sclerosis sine scleroderma and secondary antiphospholipid syndrome ACA: anti-centromere antibody; aCL: anticardiolipin antibodies; ANA: antinuclear antibodies; anti-β2GPI: anti-β2 glycoprotein I antibodies; APS: antiphospholipid syndrome; DU: digital ulcers; F: female; IgG: immunoglobulin G; LA: lupus anticoagulant; M: male; RP: Raynaud’s phenomenon; ssSSc: systemic sclerosis sine scleroderma

Authors	Age and gender	ssSSc clinical manifestations and diagnostic findings	APS clinical manifestations	Autoantibodies	Temporal relationship	Treatment	Outcome
Leite and de Carvalho [12]	48, F	RP, facial telangiectasia, arthralgia, gastroesophageal reflux; scleroderma pattern on nailfold capillaroscopy; subtle centrilobular ground-glass opacities bilaterally; diffuse oesophageal ectasia	Bilateral central retinal vein thrombosis	ANA 1:320, centromere pattern; ACA; aCL IgG; LA	APS manifestations and diagnosis preceded ssSSc features	Enalapril, omeprazole, bromopride, warfarin	No relapse or complications observed at five-year follow-up
Rossi et al. [13]	34, M	RP, facial telangiectasia, arthralgia, dyspepsia, dysphagia, asthenia; scleroderma pattern on nailfold capillaroscopy; reduced oesophageal peristaltic pressure, lower oesophageal sphincter dysfunction, grade 2 oesophagitis	Recurrent deep venous thrombosis	ANA 1:640, centromere pattern; ACA; anti-β2GPI IgM	APS manifestations preceded ssSSc features; APS was formally diagnosed after ssSSc	Nifedipine, proton pump inhibitors (unspecified), warfarin	No relapse or complications observed at two-year follow-up
Alghamdi and Derbes [14]	59, F	Gastroesophageal reflux disease, dysphagia, persistent gastrointestinal bleeding; gastric and duodenal angioectasias	Recurrent arterial thrombosis of both legs	ANA 1:640, centromere pattern; ACA; anti-RNA polymerase III; aCL IgG; anti-β2GPI IgG; LA	APS manifestations and diagnosis preceded ssSSc features	Argon plasma coagulation, octreotide, hydroxychloroquine, antiplatelet therapy (unspecified)	No relapse or complications observed at two-year follow-up
Our case	20, F	RP, multiple DU, telangiectasias, inflammatory arthralgia, dyspepsia, epigastric pain; scleroderma pattern on nailfold capillaroscopy; mild chronic gastritis	Pulmonary embolism with lingula infarction	ANA 1:2560, centromere pattern; ACA; anti-Ro52; anti-β2GPI IgG; LA	APS diagnosed after establishing ssSSc	Nifedipine, pentoxifylline, intravenous iloprost, sildenafil, bosentan, acetylsalicylic acid, heparin, followed by warfarin	DU healed in two months; four minor isolated uncomplicated DU over 20 months of follow-up; no further thrombosis or organ involvement

The first published case report [12] describes a 48-year-old female patient who initially presented with bilateral central retinal vein thrombosis and was subsequently diagnosed with APS after two positive measurements of anticardiolipin antibody (aCL) IgG and LA more than 12 weeks apart. Treatment with warfarin was initiated. Remarkably, her initial immunological workup revealed positive antinuclear antibody (ANA) with a 1:320 centromere pattern and positivity for ACA. Approximately one year later, the patient developed RP, facial telangiectasia, acute polyarthralgia, and gastroesophageal reflux. Nailfold capillaroscopy demonstrated a scleroderma pattern. A chest CT scan showed subtle centrilobular ground-glass opacities bilaterally and diffuse oesophageal ectasia, which was confirmed on oesophagography. Based on these findings, a diagnosis of ssSSc was made, and the patient was started on treatment with enalapril, omeprazole, and bromopride, resulting in clinical improvement and no further complications during a five-year follow-up period.

The second published case report [13] describes a 34-year-old male patient with a history of recurrent deep venous thrombosis. He developed RP, facial telangiectasia, arthralgia, dyspepsia, dysphagia, and asthenia. Laboratory investigation revealed a positive ANA with a 1:640 centromere pattern, positive ACA, and anti-β2GPI IgM antibodies. Nailfold capillaroscopy showed a scleroderma pattern. Oesophagogastroduodenoscopy revealed lower oesophageal sphincter dysfunction with grade 2 oesophagitis, and a high-resolution oesophageal manometry confirmed a reduction in peristaltic pressures. Based on these findings, a diagnosis of ssSSc was established. The patient started nifedipine for RP and a proton pump inhibitor, which provided relief for the gastroesophageal reflux symptoms. The recurrent deep vein thrombosis and the positive anti-β2GPI on two occasions more than 12 weeks apart led to the diagnosis of APS, and the patient was started on warfarin. Over the two-year follow-up period, the patient did not experience any additional complications.

The third published case report [14] describes a 59-year-old African American female with a history of tobacco use, hepatitis C infection, mitral valve stenosis, and recurrent arterial thrombosis of both legs, for which she underwent stenting and was placed on dual antiplatelet therapy. Laboratory investigation revealed high triple-positive aPL on two occasions more than 12 weeks apart, leading to a diagnosis of APS. Four years later, the patient developed mild symptoms of gastroesophageal reflux disease and dysphagia, along with persistent gastrointestinal bleeding. Endoscopic studies revealed gastric and duodenal angioectasias, which were treated with argon plasma coagulation and octreotide therapy. Laboratory assessment showed a positive ANA with a 1:640 centromere pattern and positive ACA and anti-RNA polymerase III antibodies. The diagnosis of ssSSc was assumed, and the patient was started on hydroxychloroquine 400 mg/day. Given the high risk of bleeding, antiplatelet therapy was maintained over anticoagulation for APS management. Over a two-year follow-up period, no further thrombotic episodes or SSc-related symptoms were observed.

In our case, recurrent DU represented the main clinical challenge, proving refractory to standard therapies. These included optimized oral vasodilators (a slow-release calcium-channel blocker, a phosphodiesterase type 5 inhibitor, and a prostacyclin analogue), adjuvant antiplatelet therapy, and an endothelin receptor antagonist as a preventive measure to reduce the incidence of new DU. However, following the thrombotic event and initiation of anticoagulation therapy, the ulcers showed a significant improvement, with complete healing and only mild, isolated recurrences over a 20-month follow-up, possibly related to difficulties in maintaining stable anticoagulation levels. Retrospectively, it can be postulated that the underlying APS may have contributed to the pathogenesis of the ulcers, potentially explaining their refractoriness to standard treatment.

The pulmonary embolism was likely multifactorial, with SARS-CoV-2 infection acting as a possible trigger due to its recognized prothrombotic potential - although this effect is typically more pronounced in severe cases requiring intensive care and cardiorespiratory support [15]. The presence of underlying APS, together with SSc-associated vasculopathy, likely potentiated the risk of pulmonary embolism.

Compared with previously reported cases, our patient presented at a younger age, consistent with EUSTAR data showing that ssSSc patients with DU tend to be younger [6]. ACA positivity in all cases and oesophageal involvement in the three earlier reports - defined by clinical symptoms and/or complementary investigations - also align with the referred registry [6].

In our case, ssSSc features were evident prior to the thrombotic events, leading to the diagnosis of ssSSc before APS was identified. In contrast, in the earlier case reports, APS manifestations preceded the development of SSc features, although APS was formally diagnosed after ssSSc in the second case [13].

No associations between SSc-related clinical manifestations and APS have been reported, apart from a higher frequency of anti-topoisomerase I (anti-Scl-70) positivity in SSc patients with secondary APS compared to those with isolated SSc [11].

Several studies have found statistically significant associations between aPL positivity and various SSc clinical features - some have reported a link between aPL and the development of DU [11,16], with anti-β2GPI identified as an independent risk factor for active DU in both univariate and multivariate analysis [11]; one study reported a link with interstitial lung disease, where elevated aCL IgM and IgG levels were associated with increased risk [16]; the same study found an association between RP and positive aCL IgM [16]; higher aCL titres were associated with an increased risk of venous thrombosis [17]. In contrast, other studies have failed to demonstrate significant associations between aPL positivity and SSc-related clinical features [18,19].

However, it is important to note that most of these studies involved relatively small patient samples, which can result in insufficient statistical power and contribute to the heterogeneous findings, thereby precluding firm conclusions. Moreover, in most of these studies, aPL measurements were not repeated after the recommended 12-week interval, as required to confirm the diagnosis of APS. Furthermore, the aPL titres observed in these studies have often been below the diagnostic thresholds for APS.

Based on the discussed data and its limitations, the current evidence does not justify routine screening for aPL in SSc. Exceptions may include situations such as pregnancy planning, recurrent thromboembolic events, or DU refractory to optimized therapy, where an underlying APS may play a contributory role [9,20].

This case report contributes to the literature by documenting a rare presentation of ssSSc associated with secondary APS, complicated by DU and pulmonary embolism. It highlights the importance of early recognition and integrated management, providing clinicians with practical insights into diagnostic strategies and therapeutic decisions.

## Conclusions

SSc is a complex disease, with DU representing one of its most challenging and debilitating manifestations. In cases of recurrent, treatment-refractory DU, the association with APS should be suspected, and appropriate screening should be performed, particularly if additional thrombotic features are present, as early identification and targeted therapy may significantly improve patient outcomes.

While the association between SSc and APS remains incompletely understood, further research is essential to clarify the underlying mechanisms, their impact on disease presentation and progression, and the most effective treatment strategies. A better understanding of this association could lead to improved diagnostic accuracy and enable more targeted therapeutic approaches.
